# An Innovative Method to Analyze the Hydraulic Fracture Reopening Pressure of Hot Dry Rock

**DOI:** 10.3390/ma16031118

**Published:** 2023-01-28

**Authors:** Deng-Deng Zhuang, Tu-Bing Yin, Zong-Xian Zhang, Adeyemi Aladejare, You Wu, Yang Qiao

**Affiliations:** 1 School of Resources and Safety Engineering, Central South University, Changsha 410083, China; 2 Oulu Mining School, University of Oulu, 90015 Oulu, Finland

**Keywords:** hot dry rock, hydraulic fracturing, reopening pressure, real-time high-temperature, fracture mechanics

## Abstract

This paper focuses on a new test method and theoretical model for measuring and evaluating the reopening pressure during hot dry rock hydraulic fracturing. Firstly, rock blocks of four lithologies were collected from the hot dry rock strata. Hydraulic fracturing tests at high temperatures in real-time were conducted using drilled cubic specimens and drilled cubic specimens with a pre-crack. Breakdown pressure, reopening pressure, and fracture toughness were measured, respectively. In addition, Brazilian splitting tests at high temperatures in real-time were performed using Brazilian disc specimens to measure tensile strength. Secondly, an empirical equation for evaluating the reopening pressure during hot dry rock secondary fracturing was developed based on fracture mechanics and hydraulic fracturing theory. Third, the values calculated by the new equation, considering breakdown pressure, fracture toughness, and tensile strength, were compared to the values determined by the classical equation and to measurement results. It was found that the new equation predicted closer reopening pressure to the measurement results, regardless of the lithology of the hot dry rock. Moreover, with increasing temperature in the specimens, the error between the value calculated by the new equation and the measurement value remained low. In contrast, the difference between the classical equation predictions and the measurement results was widened. In addition, the reopening pressure was positively correlated with tensile strength and fracture toughness. Variations in lithology and temperature affected tensile strength and fracture toughness, which then changed the hot dry rock reopening pressure.

## 1. Introduction

Hydraulic fracturing is widely used in enhanced geothermal systems (EGS). In hydraulic fracturing, a hot dry rock stratum segment in the borehole is sealed with a packer and then pressurized by fluid injection [1]. Following that, artificial cracks are generated in the dense, low-porosity hot dry rock, building a crack network that enables heat-transfer fluid to flow between injection and extraction wells [2,3]. However, if fracturing fails to create a sufficient crack count to connect the injection and extraction wells, the hot dry rock will require secondary fracturing. In secondary fracturing, the established artificial cracks continue to extend under hydraulic pressure, and the hydraulic pressure at the point of expansion is known as the reopening pressure [4].

Reopening pressure is a crucial parameter for secondary fracturing design. There are two methods to determine the reopening pressure: (1) Secondary fracturing testing at the project site or in the laboratory to monitor the pressure [5,6]; (2) Calculation of the reopening pressure based on the conventional hydraulic fracturing theoretical model [7]. Specifically, the breakdown pressure at initial fracturing equals the sum of the minimum tangential stress in the hole wall and the rock tensile strength. Assume that the stress state surrounding the constructed artificial crack, especially the minimum shear stress, remains constant as the crack continues to extend. Then the reopening pressure for crack re-extension lacks the tensile strength compared to the initial fracturing [8,9]. Therefore, the reopening pressure amounts to the difference between the breakdown pressure and the tensile strength [10]. By comparing Method 1 and Method 2 in previous studies, it can be seen that the reopening pressure calculated by the conventional theoretical model is always above the measurement result [11,12,13]. This deviation was noticed by some researchers. They found that the stress field around the existent artificial crack changed during secondary fracturing, and the continuity assumption condition in the elastoplastic theory calculation was not valid anymore, thus resulting in the error of the conventional theory equation [14,15,16,17]. For example, Jean-Pierre Petit et al. [18] found that the stress concentration at the crack tip rises with reopening, until that failure of the tip region results in branch crack formation, triggering both slip along the vein and hydraulic pressure drop. Rutqvist et al. [7] demonstrated that when the hydraulic crack reopens, the induced crack significantly disturbs the ideal case with a linear elastic, homogenous, and isotropic medium in the classical theory for hydraulic fracturing stress measurement. Therefore, the fracture mechanics theory, which explains the change in the surrounding stress field as the crack reopens, should be considered in the reopening pressure calculation model as a reference for secondary fracturing tests [19,20].

Unfortunately, up till now, the secondary fracturing reopening pressure of hot dry rock has not been widely investigated in terms of either test monitoring or theoretical calculation [21,22]. The reasons are twofold: (1) Specimens are required to be in a heated furnace to maintain a real-time high temperature in hot dry rock hydraulic fracturing tests, which limits specimen size. In previous tests, cylindrical specimens were less than 100 mm in diameter and cubic specimens were less than 300 mm in side length. As a result, the generated cracks in the hydraulic fracturing ran through the entire specimen, resulting in the impossibility of further secondary fracturing tests [23,24,25,26]; (2) The reopening pressure calculation theory based on fracture mechanics is lacking for hot dry rock hydraulic fracturing [27,28,29,30]. In conclusion, the above two factors hinder the application of hot dry rock secondary fracturing technology in EGS projects [31,32]. It is important to propose new test method and theoretical derivation to analyze the hot dry rock hydraulic fracturing.

In this paper, a new test method for measuring reopening pressure and fracture toughness during hot dry rock hydraulic fracturing, and a new theoretical model for evaluating reopening pressure based on fracture mechanics are proposed. Since the hydraulic crack runs across the specimen, resulting in the secondary fracturing inability, a pre-crack was cut to simulate the initial hydraulic crack. The threshold pressure for pre-crack extension in the hydraulic fracturing test can be defined as the secondary fracturing reopening pressure. Meanwhile, the pre-cracked specimen’s fracture toughness was measured in a real-time high-temperature hydraulic fracturing test. In addition, breakdown pressure was monitored in the real-time high-temperature hydraulic fracturing test on the non-pre-cracked specimen, and tensile strength was determined in the real-time high-temperature Brazilian splitting test. Finally, all measurement results were used to validate the new reopening pressure calculation model.

## 2. Materials and Experimental Methodology

### 2.1. Material Features and Sample Preparation

**Figure 1:**
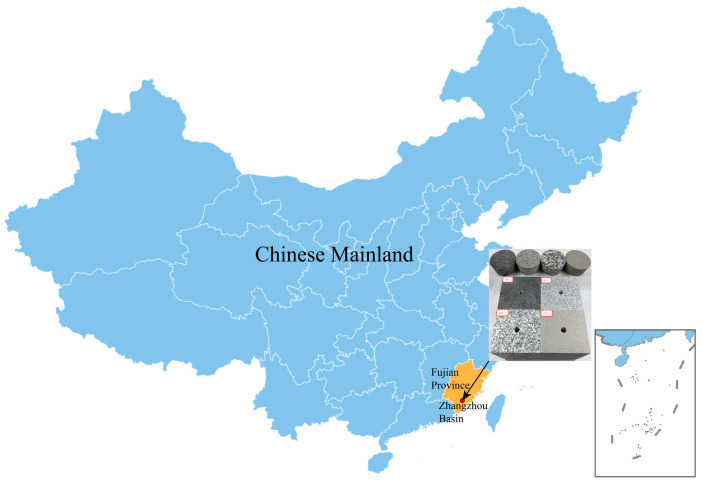
Location diagram of the rock specimens used in this study.

**Figure 2:**
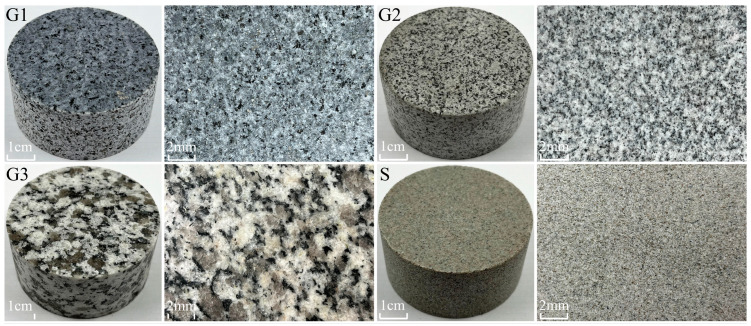
Photos and matrix surfaces of Brazilian disc specimens of the lithologies G1, G2, G3, and S.

**Figure 3:**
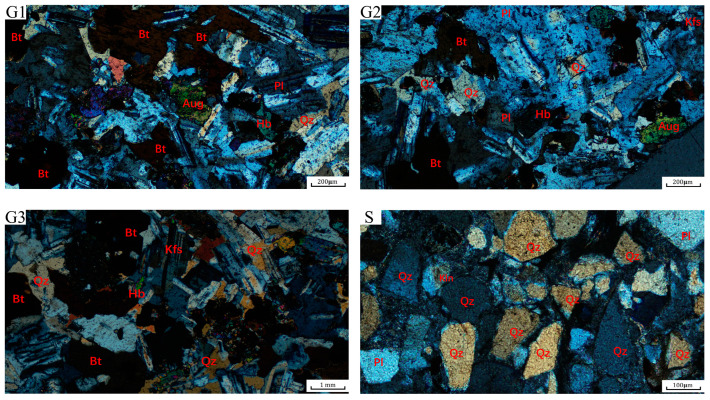
Micrographs of G1, G2, G3, and S lithological specimens by a single-polarization microscope.

**Figure 4:**
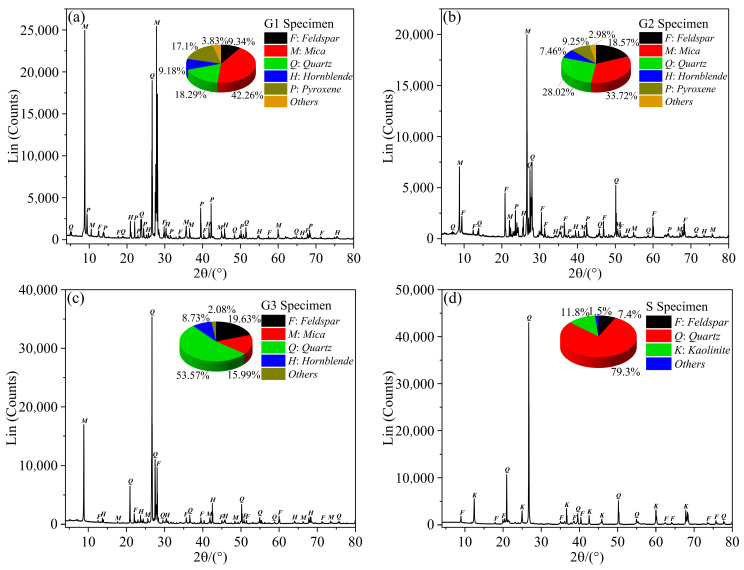
X-ray diffraction (XRD) patterns of rock specimen: (**a**) G1 specimen; (**b**) G2 specimen; (**c**) G3 specimen; (**d**) S specimen.

The Zhangzhou Basin in Fujian Province is a prominent hot dry rock geothermal exploration area on China’s eastern coast (Figure 1). Most of the hot dry rock lithologies in the area are granites, with a few sandstones [33]. To simulate hot dry rock realistically, three types of granite and one type of sandstone were collected as the test material, taken from outcrops with the same geological period of the hot dry rock stratum. Different lithologies are apparently distinct in particle size and colour, as shown in Figure 2. G1 and G2 types of granite are in a particle size range of 0.1–1.2 mm and are coloured in dark grey and light grey, respectively. G3 type of granite has a particle size range of 1.0–3 mm and is in grey-white. The sandstone has a particle size in the range of 0.05–0.25 mm and is grey-green. The mineral composition of all lithologies was analyzed using polarised micrographs of rock sections and X-ray diffraction patterns, as shown in Figure 3 and Figure 4. The main minerals in the G1 granite are feldspar (9.34%), mica (42.26%), quartz (18.29%), hornblende (9.18%), pyroxene (17.1%); in the G2 granite, they are feldspar (18.57%), mica (33.72%), quartz (28.02%), hornblende (7.46%), pyroxene (9.25%); in the G3 granite, they are feldspar (19.63%), mica (15.99%), quartz (53.57%), hornblende (8.73%), and in the sandstone they are quartz (79.3%), kaolinite (11.8%), and feldspar (7.4%).

**Figure 5:**
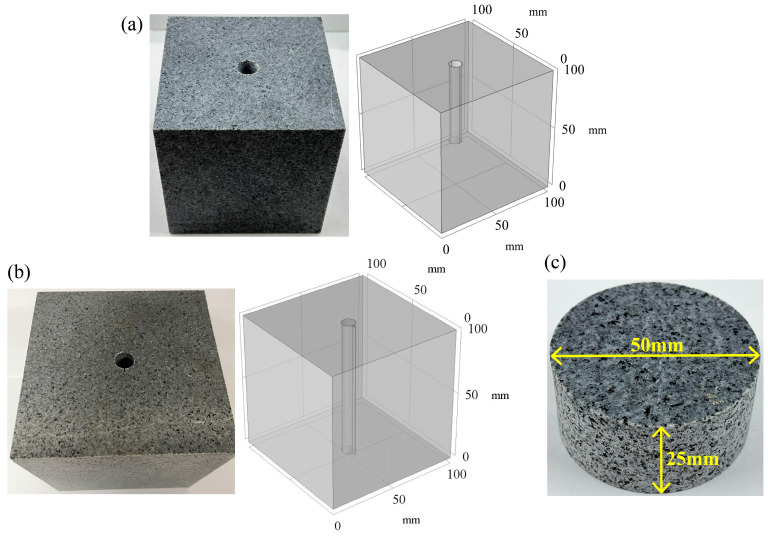
Specimen preparation: (**a**) HFS specimen for hydraulic fracturing tests; (**b**) SFS specimen for secondary fracturing tests; (**c**) BSS specimens for Brazilian splitting tests.

Each lithology was processed into three kinds of specimens for different experiments: (a) Each block was cut into cubes with sides of 100 mm × 100 mm × 100 mm, and a hole with a depth of 55 mm and a diameter of 10 mm was drilled at the surface center to conduct the hydraulic fracturing test at high temperature in real-time and monitor the breakdown pressure. (b) The rock was processed into a thick-walled hollow cube with a pre-crack, as described in our earlier study for assessing the fracture toughness of hot dry rock [23]. The specific steps were to cut a cube with sides of 100 mm × 100 mm × 100 mm, then drill a 12 mm diameter of through-hole at the center of the surface. Finally, a crack with a width of 0.1 mm, a length of 3 mm, and a height of 100 mm was cut in a direction parallel to the hole’s central axis and perpendicular to the surface. The prepared specimen was subjected to a secondary fracturing test at high temperature in real-time to determine the reopening pressure and fracture toughness. (c) Brazilian disc specimens of 50 mm diameter and 25 mm thickness were processed for tensile strength testing in real-time at high temperature. HFS, SFS, and BSS are abbreviations for hydraulic fracturing specimen, secondary fracturing specimen, and Brazilian splitting specimen, respectively, and the various types of specimens are shown in Figure 5. The ends of all specimens were flattened until parallelism was within 0.002 mm/mm. The perpendicularity of the drilled holes and pre-cut cracks was within the guidelines of the International Society of Rock Mechanics (ISRM) [34].

### 2.2. Experimental Equipments

**Figure 6:**
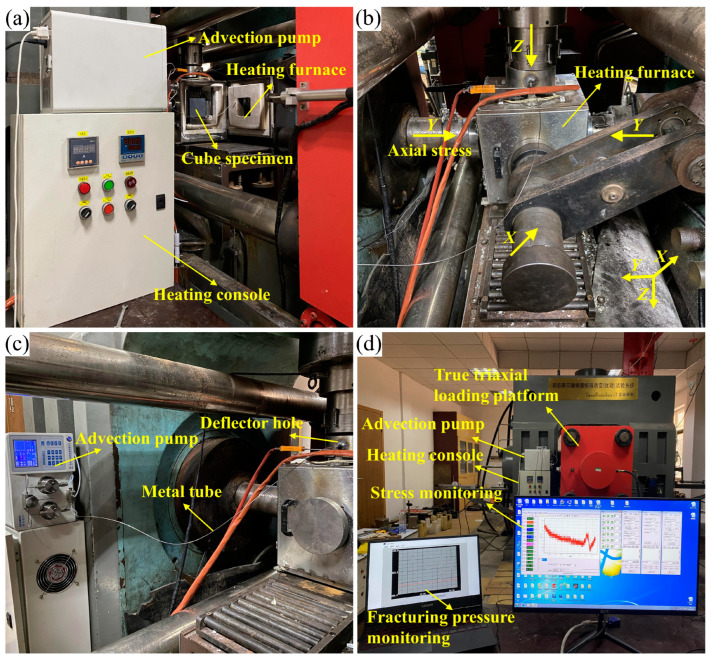
Real-time high temperature true-triaxial hydraulic fracturing platform: (**a**) the heating system; (**b**) the true triaxial loading system; (**c**) the water injection and sealing system; (**d**) the computer monitoring system.

Hydraulic fracturing tests were performed on a self-developed real-time high-temperature true triaxial hydraulic fracturing platform. As shown in Figure 6, the platform consists of four systems: a heating system, a true triaxial loading system, a water injection and sealing system, and a computer monitoring system. The heating system comprises a six-sided open-hole furnace and a heating console that enables the specimen to be heated at a rate of 1–20 °C/min up to 800 °C. In the true triaxial loading system, the loading pads are inserted into the furnace wall holes to ensure that the specimen is heated and loaded to a maximum of 3000 kN in the X, Y, and Z axes, respectively. The water injection and sealing system includes a 2 PB-00IV advection pump, a metal tube, a loading pad with an internal deflector hole, and an asbestos gasket sandwiched between the specimen surface and the loading pad. The asbestos gasket serves as a seal, and the maximum working temperature is 900 °C. The advection pump allows water pressure up to 42 MPa at injection flow rates of 0.01–10 mL/min. The fracturing fluid contains a red tracer to mark the cracks after fracturing. The computer monitoring system monitors water injection pressure and loading pressure with a resolution and accuracy of 0.1 MPa and 1%, respectively. In addition, Brazilian splitting tests were conducted on a real-time high-temperature loading platform, as shown in Figure 8.

### 2.3. Hydraulic Fracturing Test

**Figure 7:**
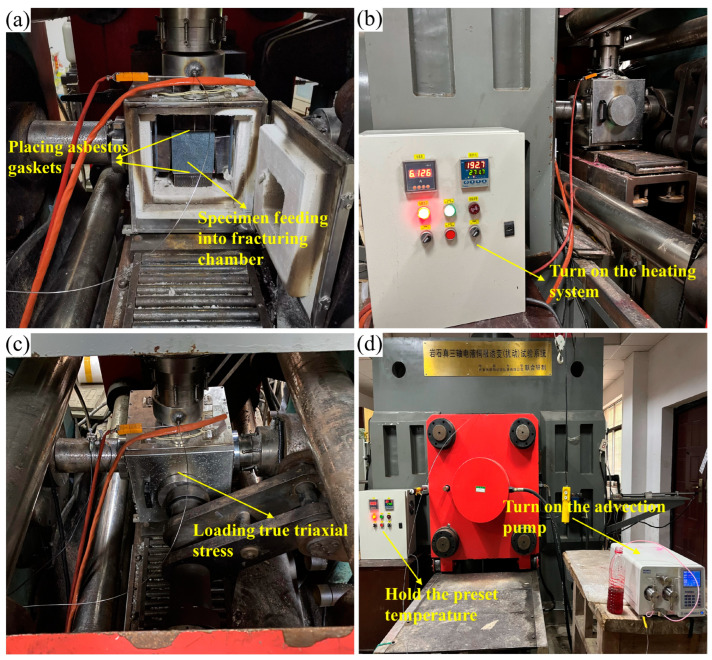
Main hydraulic fracturing procedures: (**a**) HFS or SFS specimen was placed in the fracturing chamber; (**b**) specimen was heated in the furnace; (**c**) three directions confining stresses were applied; (**d**) specimen fractured by water injection.

In the hydraulic fracturing tests, HFS and SFS specimens were used to determine the breakdown pressure and reopening pressure, respectively. There were 72 specimens in total, with 9 HFS and 9 SFS for each lithology. They were equally divided into three temperature groups of 100 °C, 300 °C and 500 °C, numbered T1, T3 and T5, each containing three specimens. The main hydraulic fracturing procedure was essentially identical for the different specimens, as shown in Figure 7. The specimens were first heated to a predetermined temperature, then loaded to a preset triaxial stress, and finally the advection pump was turned on to inject water for fracturing until the injection pressure suddenly dropped to finish the fracturing. The specific steps were as follows:

(I) Checked and connected the apparatus.
(II) Placed the specimen in a pressurized and heated chamber constructed by loading pads and the heating furnace. In the Z-axis direction, the upper loading pad contains a deflection hole inside to allow fracturing fluid to be pumped into the specimen during loading. For HFS, since the specimen hole opens at one end only, it is enough to seal the gap between the hole and the loading pad with a center-perforated asbestos gasket. For SFS, the specimen hole opens at both ends, so that besides sealing the upper loading pad, a complete asbestos pad is required to seal the gap between the specimen hole and the lower loading pad.
(III) Turned on the heating furnace. Temperature groups T1, T3, and T5 were heated to 100, 300, and 500 °C, respectively. The heating rate was maintained at 1 °C/min to avoid thermal shock damage.
(IV) Held the preset temperature and turned on the pressure pump to load the true triaxial stress. The maximum, minimum, and intermediate principal stresses loaded on the specimen were 30 MPa, 20 MPa, and 25 MPa, respectively. For SFS specimens, to ensure that the fracture extends in the pre-crack direction, the pre-crack should coincide with the preferred fracture direction (PFD) during hydraulic fracturing, which is perpendicular to the minimum principal stress and parallel to the maximum principal stress [35].
(V) Kept the preset temperature and loading pressure, then turned on the advection pump for fracturing. The pump pressure increased to a peak point and then abruptly dropped to a stable value, when fracturing was completed. The injection rate was 10 mL/min during fracturing.
(VI) Took out the specimen after pressure relief and cooling, examined the cracks on the specimen surface, and analyzed the fracturing curve.

### 2.4. Brazilian Test

**Figure 8:**
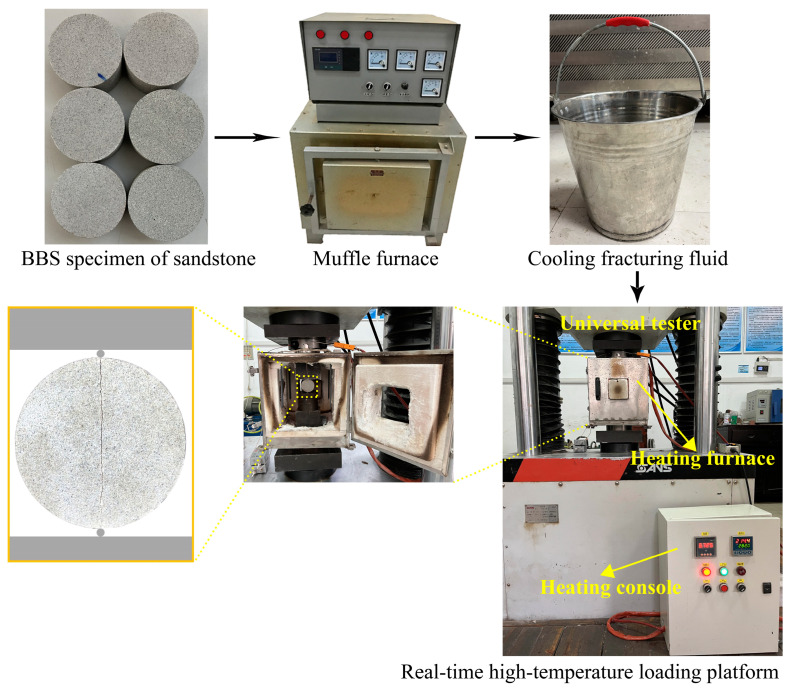
Brazilian splitting test procedures.

Figure 8 shows the main process of the Brazilian splitting test. A total of 36 BSS specimens were tested, nine for each lithology and equally divided into three temperature groups of 100 °C, 300 °C and 500 °C, numbered T1, T3 and T5, each containing three specimens. Prior to the test, the specimen being cooled by the fracturing fluid during hydraulic fracturing at high temperature in real-time was reproduced. Specifically, the BSS specimens were heated to the preset temperature at a rate of 1 °C/min, then cooled in a bucket with fracturing fluid for 3 min. This is due to the approximate duration of 3 min from water injection to fracturing completion in Section 2.3. In the test, the specimen was held at the preset temperature on the real-time high-temperature loading platform, and axial pressure was applied along the diameter at a loading rate of 0.06 mm/min until a break occurred.

## 3. Experimental Results

### 3.1. Breakdown and Reopening Pressures of Hot Dry Rock in Hydraulic Fracturing

**Figure 9:**
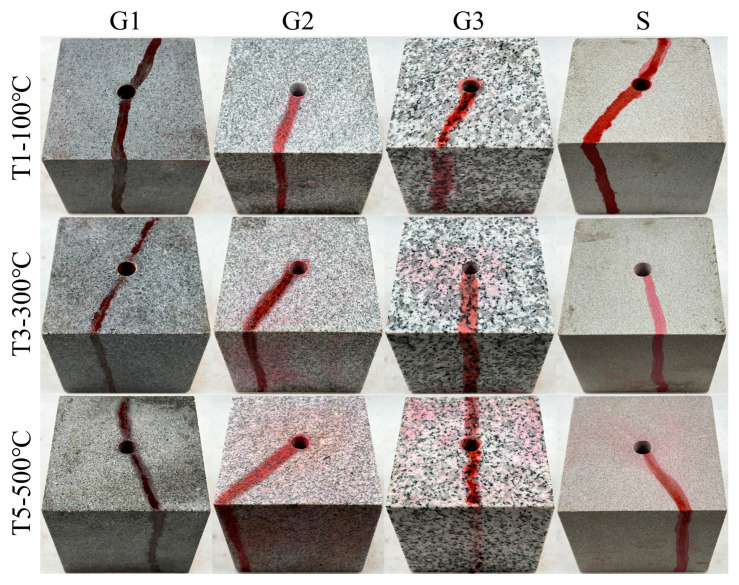
Photograph of HFS specimens of G1, G2, G3, and S lithologies after fracturing.

**Figure 10:**
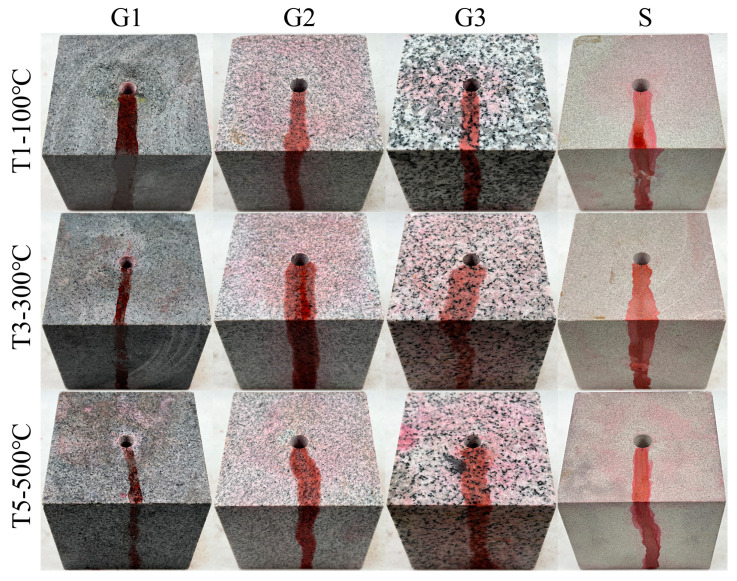
Photograph of SFS specimens of G1, G2, G3, and S lithologies after fracturing.

**Figure 11:**
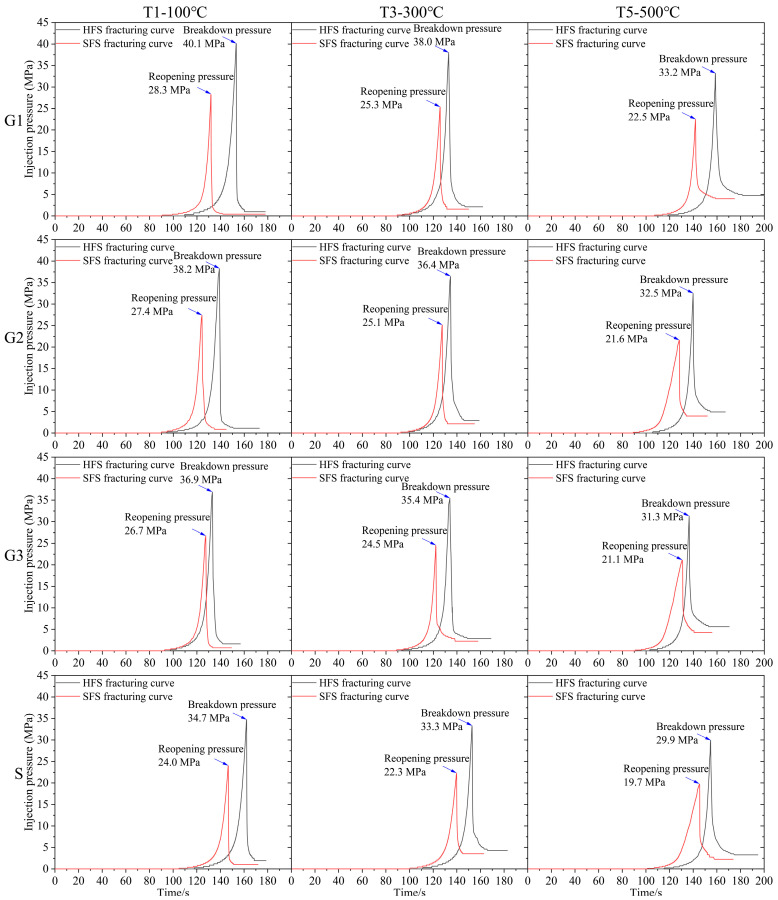
Fracturing curves for HFS and SFS specimens of four lithologies at different temperatures.

Figure 9 and Figure 10 display the photos of the HFS and SFS specimens from G1, G2, G3, and S lithologies, respectively, after being fractured. The hydraulic crack was tagged by a red tracer in the fracturing fluid. Figure 11 depicts the fracturing curves for the HFS and SFS specimens from the four lithologies. It can be seen that the fracturing curves for the HFS and SFS specimens were extremely similar, all ranging from a slow to abrupt increase and then a sharp fall after the specimen was broken. The peak points of the two types of fracturing curves represent the breakdown pressure and pre-crack reopening pressure, respectively. The breakdown pressure and reopening pressure of the HFS and SFS specimens at different temperatures are listed in Table 1 and Table 2, respectively. The data show that the specimen breakdown pressure was larger than the reopening pressure, and that both pressures decreased with increasing temperature.

### 3.2. Fracture Toughness of Hot Dry Rock in Hydraulic Fracturing

The fracture toughness of high-temperature rock during hydraulic fracturing can be calculated based on the reopening pressure and the associated fracture mechanics model. By superimposing the uniform tension stress intensity factor of the edge-notched plate and the uniform pressure at the crack plane of the edge-notched plate, Clifton et al. [36] developed a fracture toughness calculation model for thick-walled hollow cylindrical specimens with pre-cracking subjected to internal hydraulic pressure and conducted the earliest tests to measure fracture toughness by hydraulic fracturing. However, due to the non-uniform distribution of the tangential stress on the borehole wall in a cubic specimen under true triaxial stress as opposed to a cylindrical specimen, this calculation model is not available in this study [37,38]. In an alternative approach, we found that Abou-Sayed et al. [39] investigated the pre-crack extension in the borehole at an arbitrary angle to the principal stress direction, and determined a fracture toughness calculation model related to the borehole pressure, principal stress, and pre-crack length. Specifically, for the pre-crack parallel maximum principal stress σ_1_ and perpendicular minimum principal stress σ_3_, they propose a calculation equation for the reopening pressure *P*_r_ at pre-crack extension.

(1) $$P_{r}=\frac{1}{2}\left(3\sigma_{3}-\sigma_{1}+\frac{K_{IC}}{0.6\sqrt{\pi L}}\right)$$

where *K_IC_* represents the pre-crack re-extension stress intensity factor, also defined as fracture toughness; *L* is the pre-crack length.

**Figure 12:**
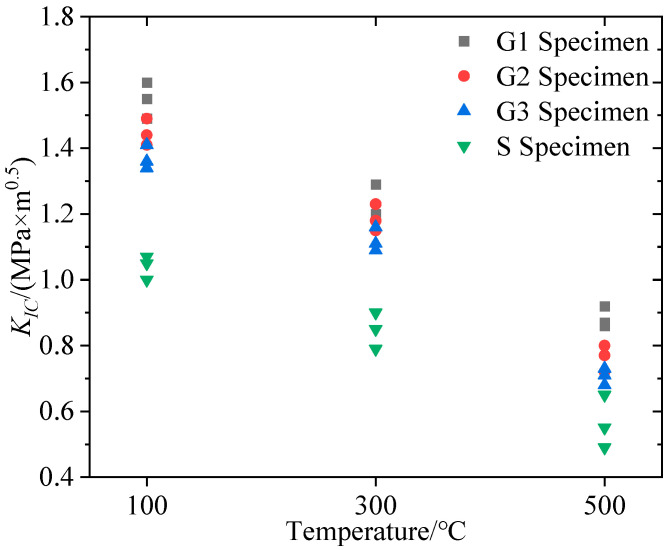
Variation of fracture toughness with specimen lithology and temperature.

The fracture toughness calculated from the pre-crack reopening pressure and Equation (1) is listed in Table 1. As shown in Figure 12, all lithological specimens’ fracture toughness decreased with increasing temperature during hydraulic fracturing at high temperature in real-time. In each temperature group, the fracture toughness of the four lithology specimens was in the order of G1 > G2 > G3 > S.

### 3.3. Tensile Strength from Brazilian Test

The tensile strength *σ_t_* in the Brazilian splitting test can be calculated as follows [40]:

(2) $$\sigma_{t}=2P_{\max}/\pi Dt$$

where *P*_max_ is the peak loading pressure, *D* is the disc diameter, and *t* is the disc thickness.

**Figure 13:**
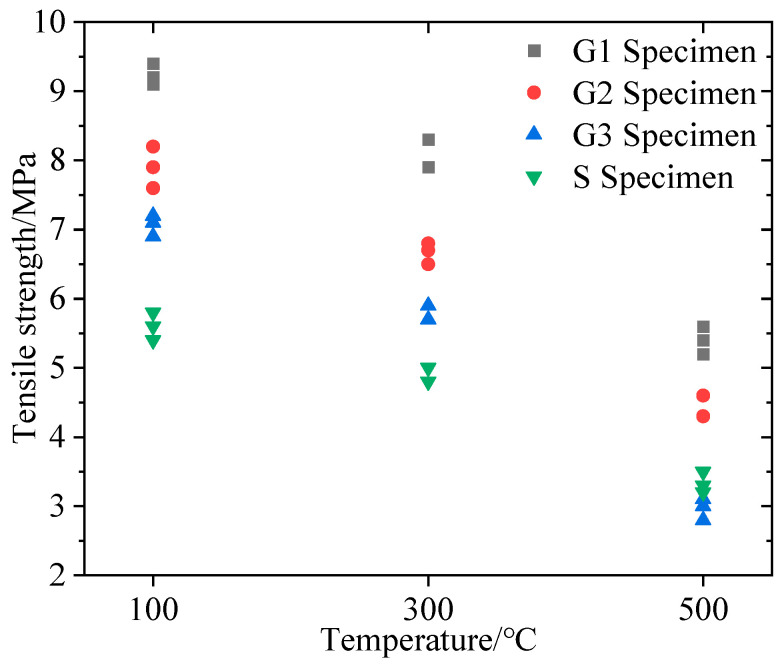
Variation of tensile strength with specimen lithology and temperature.

The tensile strength measurement results are listed in Table 1. As shown in Figure 13, the tensile strength of all lithological specimens decreased as the temperature rose. In each temperature group, the tensile strength of the four lithological specimens was in the order of G1 > G2 > G3 > S.

## 4. Calculation Model for Hydraulic Fracturing Reopening Pressure

### 4.1. Calculation Model

The conventional reopening pressure calculation model was originally derived from the hydraulic fracturing geo-stress measurement method proposed by Hubbert et al. [41]. The measuring method initially plugs the upper and lower ends of the measurement borehole segment and then pumps a high-pressure fluid into the strata around the borehole wall to induce a hydraulic crack. As the tensile strength is less than the shear strength and compressive strength, the hydraulic crack can be triggered when the water pressure equals the tensile strength plus the minimum shear stress of the borehole wall surrounding rock, which fulfills the following equation [42]:

(3) $$P_{b}=3\sigma_{h}-\sigma_{H}+T$$

where *σ*_h_ represents the minimum principal stress, and *σ*_H_ represents the maximum principal stress.

Due to the tensile failure that has occurred, when the measured strata segment is secondary to fracturing, the reopening pressure *P*_r_ that allows the hydraulic crack to re-extend is [42]:

(4) $$P_{r}=3\sigma_{h}-\sigma_{H}$$

The reopening pressure *P*_r_ can be calculated from Equations (3) and (4) as:

(5) $$P_{r}=P_{b}-T$$

It is obvious that the conventional calculation model is based on the hypothesis that the surrounding stress field and the minimum shear stress remain constant after the hydraulic crack re-extends, thus the reopening pressure only lacks tensile strength compared to the breakdown pressure [43,44]. However, this hypothesis is contrary to fracture mechanics theory, where the stress field around the hydraulic crack is inevitably altered as it re-extends [45,46]. Therefore, it is not valid to calculate the reopening pressure using the continuous assumptions of the elastic-plastic theory, and a new calculation model based on fracture mechanics should be developed.

Prior to theory derivation, it was widely acknowledged that only tensile failure occurs in the pre-crack under hydraulic fracturing, which is a classic type I fracture [47]. According to the infinite thin panel theory of fracture mechanics, the equation for the type I fracture stress intensity factor for the crack of length a is [48]:

(6) $$K_{I}=\sqrt{\pi a}\int_{-a}^{a}\sigma_{y}\left(x,0\right){\left(\frac{a+x}{a-x}\right)}^{1/2}dx$$

where *σ_y_* is the stress in the y-direction.

**Figure 14:**
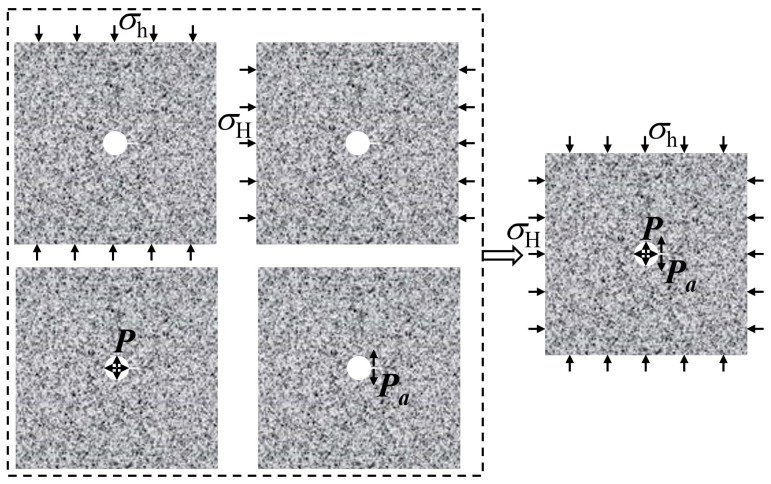
Superposition of four kinds of fracture type.

In the hydraulic fracturing test, the SPS specimen fracture pattern can also be superimposed by the four fracture types indicated in Figure 14.

The stress intensity factors for each of the four independent module loadings are as follows [15,49]:

(7) $$K_{I}\left(\sigma_{h}\right)=-\sigma_{h}\sqrt{R_{0}}g\left(b\right)$$

(8) $$K_{I}\left(\sigma_{H}\right)=-\sigma_{H}\sqrt{R_{0}}f\left(b\right)$$

(9) $$K_{I}\left(P\right)=P\sqrt{R_{0}}h_{0}\left(b\right)$$

(10) $$K_{I}\left(P_{a}\right)=P\sqrt{R_{0}}h_{a}\left(b\right)$$

(11) $$g\left(b\right)={\left[\pi \left(1+a/R_{0}\right)\right]}^{1/2}\left[1-\frac{2}{\pi }\sin^{-1}\frac{1}{\left(1+a/R_{0}\right)}\right]+2\left[{\left(1+a/R_{0}\right)}^{2}+1\right]{\left[\frac{{\left(1+a/R_{0}\right)}^{2}-1}{\pi {\left(1+a/R_{0}\right)}^{7}}\right]}^{1/2}$$

(12) $$f\left(b\right)=-2{\left\{\left[{\left(1+a/R_{0}\right)}^{2}-1\right]/\left[\pi {\left(1+a/R_{0}\right)}^{7}\right]\right\}}^{1/2}$$

(13) $$h_{0}\left(b\right)=1.3\times \frac{a/R_{0}}{1+{\left(1+a/R_{0}\right)}^{3/2}}+7.8\times \frac{\sin\left(a/2R_{0}\right)}{2{\left(1+a/R_{0}\right)}^{5/2}-1.7}$$

(14) $$h_{a}\left(b\right)={\left[\pi \left(1+a/R_{0}\right)\right]}^{1/2}\left(1-\frac{2}{\pi }\sin^{-1}\frac{1}{1+a/R_{0}}\right)$$

By superimposing Equations (7)–(10) equivalently to Equation (6), the equation for the reopening pressure of the SFS specimen when the pre-crack re-extends is as follows:

(15) $$P_{r}=\frac{1}{h_{0}\left(b\right)+h_{a}\left(b\right)}\left(\frac{K_{IC}}{\sqrt{R_{0}}}+\sigma_{h}g\left(b\right)+\sigma_{H}f\left(b\right)\right)$$

where *K_IC_* represents the fracture toughness of the type I fracture. Equation (15) reflects that the reopening pressure *P*_r_ is related to the pre-crack length, fracture toughness, and stress values rather than just the breakdown pressure and tensile strength as in Equation (5).

Equation (16) is equal to Equation (3) minus Equation (15):

(16) $$P_{r}=\frac{1}{h_{0}\left(b\right)+h_{a}\left(b\right)}\left(\frac{K_{IC}}{\sqrt{R_{0}}}+\sigma_{h}g\left(b\right)+\sigma_{H}f\left(b\right)\right)$$

For the SFS specimens in this study, with a = 0.5R, Equation (16) can be simplified to:

(17) $$P_{b}-P_{r}=1.86\sigma_{h}-0.84\sigma_{H}+T-\frac{K_{IC}}{1.9\sqrt{R_{0}}}$$

or

(18) $$P_{r}=P_{b}-1.86\sigma_{h}+0.84\sigma_{H}-T+\frac{K_{IC}}{1.9\sqrt{R_{0}}}$$

According to Equation (18) from fracture mechanics, the reopening pressure depends on the breakdown pressure and tensile strength, but also on the fracture toughness, the stress field, and the borehole radius.

### 4.2. Validation of the Proposed Calculation Model

In Section 4.1, a new equation was developed for calculating the reopening pressure of a pre-crack specimen during hydraulic fracturing. To verify the accuracy of the new equation relative to the classical equation, the reopening pressure calculations of Equations (5) and (18) were compared with the measurement results, respectively.

A total of 108 HFS, SFS, and BSS specimens were tested for hydraulic fracturing, secondary fracturing, and Brazilian splitting, respectively. Based on the breakdown pressure, fracture toughness, tensile strength, stress values, and borehole radius obtained from each test, different reopening pressure values were calculated by Equations (5) and (18), as listed in Table 2.

**Figure 15:**
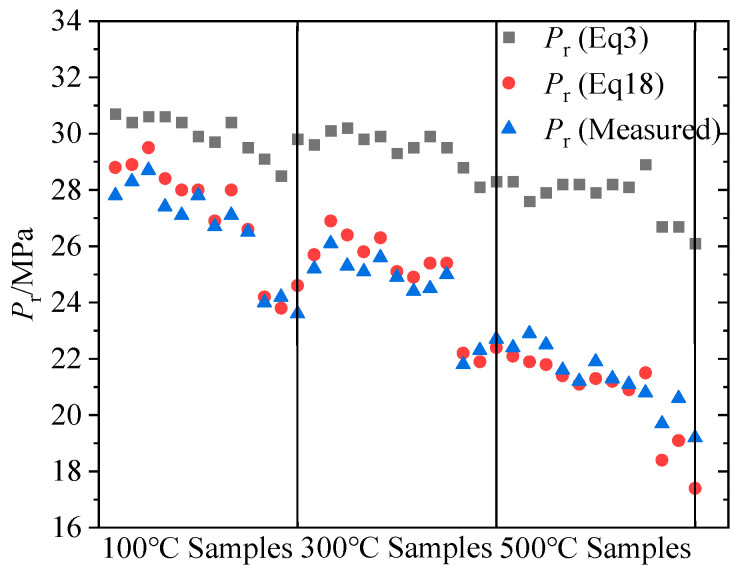
Variation of measured and calculated reopening pressure with temperature for all lithological samples.

Figure 15 depicts the reopening pressure of all lithological samples at 100 °C, 300 °C, and 500 °C. The reopening pressure calculated in Equation (5) for each lithology and temperature specimen was generally larger than the measurement result in the SFS specimen hydraulic fracturing, and this trend became more evident as sample temperature increased. In contrast, the reopening pressure calculated in Equation (18) for each lithology and temperature specimen was extremely close to the measurement result. This illustrates that the new reopening pressure calculation model developed from fracture mechanics theory is more accurate than the conventional calculation model for hot dry rock with various lithologies and temperatures.

## 5. Discussion

### 5.1. Feasibility of the Novel Analysis Method for Hot Dry Rock Reopening Pressure

**Figure 16:**
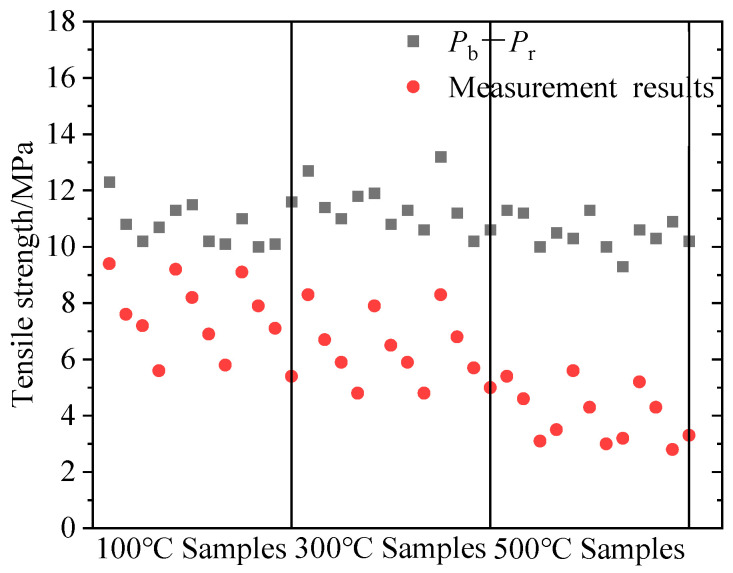
Variation of measured and calculated tensile strength with temperature for all lithological samples.

In conventional hydraulic fracturing theory, the tensile strength equals the breakdown pressure minus the reopening pressure. However, the tensile strength calculated based on the measured breakdown pressure and reopening pressure of the HFS and SFS specimens was obviously larger than the measurement results of the Brazilian splitting at high temperature in real-time, as shown in Figure 16. This discrepancy is prevalent in previous studies, indicating that the formulation based on conventional hydraulic fracturing theory is deficient.

In comparison, for all lithologies and temperatures, the reopening pressures calculated by the new theoretical model derived from fracture mechanics theory were closer to the measurement results of the SFS specimen hydraulic fracturing, indicating that the new calculation method is more compatible with engineering practice. In addition, the gap between the reopening pressure calculated in Equation (5) and the measurement results became more apparent as the temperature increased, as shown in Figure 15. The reason is that the reduction rate of tensile strength was faster than the reduction rate of breakdown pressure as the temperature rose, resulting in an ever higher computation of Equation (5) compared to the measured results. In summary, as the specimen temperature increased for each lithology type, the difference between the reopening pressure calculated by the new theoretical model and the measurement results remained constant, whereas the error between the calculation of the conventional equation and the measurement results increased gradually. Therefore, the theoretical model derived from fracture mechanics is more realistic to compute the reopening pressure for hot dry rock with high temperature and various lithological features, especially as the strata temperature increases with drilling depth.

### 5.2. Influence Factors of Hot Dry Rock Reopening Pressure

**Figure 17:**
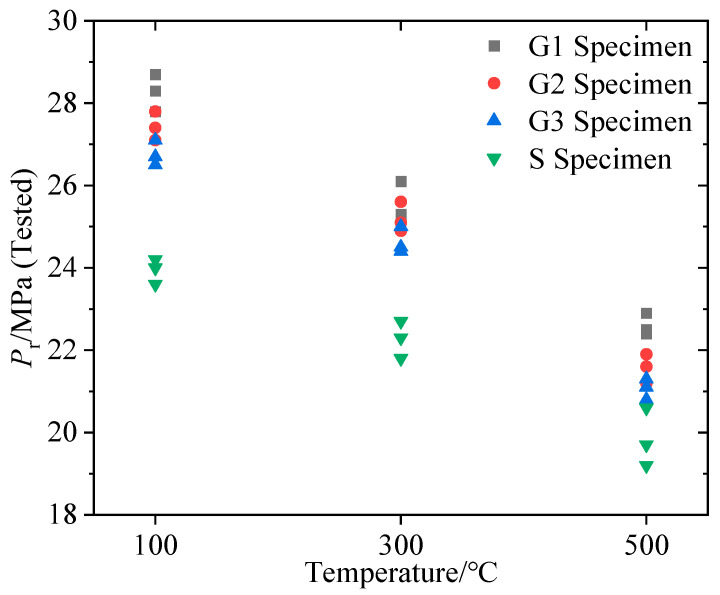
Variation of measured reopening pressure with specimen lithology and temperature.

**Figure 18:**
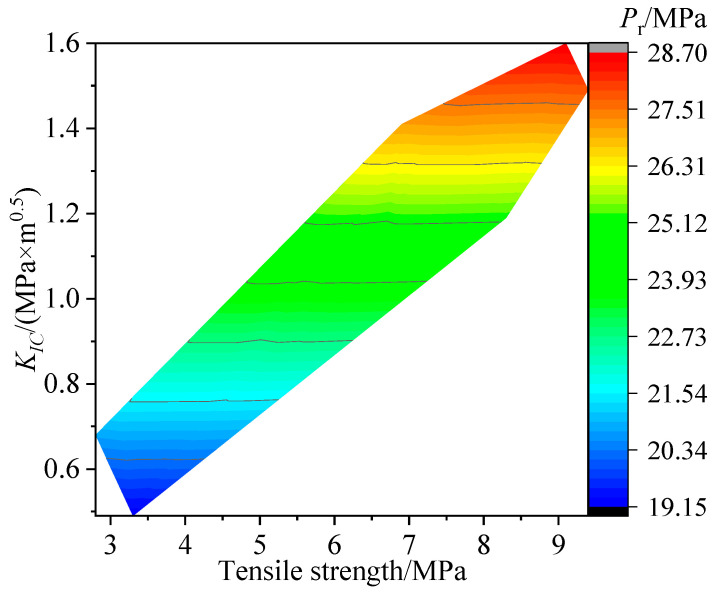
Variation of measured reopening pressure with fracture toughness and tensile strength.

Based on the reopening pressure tested from the SFS specimen hydraulic fracturing, it can be found that the hot dry rock reopening pressure closely depends on the lithology and temperature, as shown in Figure 17. Both laws can be summarized as follows: (1) The hot dry rock reopening pressure decreased with increased temperature for all lithologies; (2) the reopening pressure was G1 > G2 > G3 > S in descending order by lithology type in each temperature group. It can be assumed that the above laws are due to the correlation between the reopening pressure and the variation in hot dry rock tensile strength and fracture toughness. As shown in Figure 12 and Figure 13, the fracture toughness and tensile strength declined with increased temperature for all lithology specimens and were ordered by lithology as G1 > G2 > G3 > S in each temperature group. This suggests that the evolution of reopening pressure, tensile strength, and fracture toughness with hot dry rock lithology and temperature are identical. Furthermore, the reopening pressure of the specimens correlates well with tensile strength and fracture toughness, as shown in Figure 18. Equation (19) is the multivariate fitted equation relating reopening pressure, tensile strength, and fracture toughness in Figure 18; R^2^ = 9.999. This demonstrates a good correlation between the reopening pressure and the tensile strength and fracture toughness of the hot dry rock. Overall, the temperature and lithology of hot dry rock not only affect tensile strength and fracture toughness but furthermore result in variations in reopening pressure.

*P*r = 15 − 0.00567T + 8.61806*K_IC_* (19)

## 6. Conclusions

In this paper, real-time high-temperature hydraulic fracturing and Brazilian splitting tests on HFS, SFS and BSS specimens of four lithologies were performed to measure the breakdown pressure, reopening pressure, fracture toughness and tensile strength of hot dry rock, respectively. Furthermore, an innovative equation for calculating the hot dry rock reopening pressure was developed based on fracture mechanics. To validate the new equation, the reopening pressure calculated by the new equation and that by the conventional equation were compared with the hydraulic fracturing test result from SFS specimens. Finally, the feasibility of the new equation and the influence factors on the hot dry rock reopening pressure were analyzed, and the main conclusions are as follows:

1. The new test and theoretical method for determining the reopening pressure was applicable to hot dry rock with different lithologies and temperatures. Compared to the conventional equation, the reopening pressure calculated by the new equation was closer to the measurement result. Moreover, as the specimen temperature rose, the deviation between the reopening pressure calculated by the conventional equation and that from the tests became larger, while the calculation of the new equation was consistently close to the measurement result.
2. The reopening pressure of hot dry rock correlated well with the tensile strength and fracture toughness of the rock tested, showing that the higher the tensile strength and fracture toughness, the larger the reopening pressure during secondary fracturing. In other words, the specimen’s lithology and temperature affect its tensile strength and fracture toughness, and change its reopening pressure with the same regularity.
3. Hot dry rock reopening pressure is strictly dependent on breakdown pressure, tensile strength, fracture toughness, geostress, borehole radius, and initial hydraulic crack length, rather than just breakdown pressure and tensile strength as defined by the conventional theoretical model. Therefore, such critical parameters as breakdown pressure, tensile strength, fracture toughness, geostress, borehole radius, and initial hydraulic crack length that affect reopening pressure should be considered in the design of hot dry rock secondary fracturing.

## Figures and Tables

**Table 1:** Breakdown pressure, fracture toughness and tensile strength of specimens at various temperatures and lithologies.

T/°C	Type	*P*_b_/MPa				*K_IC_*/(MPa × m^0.5^)				T/MPa			
		No.1	No.1	No.1	Ave.	No.1	No.1	No.1	Ave.	No.1	No.1	No.1	Ave.
100	G1	40.1	39.6	39.7	39.8	1.49	1.55	1.60	1.55	9.4	9.2	9.1	9.2
	G2	38.2	38.6	37.8	38.2	1.44	1.41	1.49	1.45	7.6	8.2	7.9	7.9
	G3	36.9	37.3	36.6	36.9	1.36	1.41	1.34	1.37	7.2	6.9	7.1	7.1
	S	34.7	34.3	35.2	34.7	1.05	1.07	1.00	1.04	5.6	5.8	5.4	5.6
300	G1	37.9	38.0	38.5	38.1	1.19	1.29	1.20	1.23	8.3	7.9	8.3	8.2
	G2	36.5	36.4	36.1	36.3	1.18	1.23	1.15	1.19	6.7	6.5	6.8	6.7
	G3	35.4	35.8	35.2	35.5	1.09	1.11	1.16	1.12	5.9	5.9	5.7	5.8
	S	33.6	32.9	33.3	33.3	0.79	0.85	0.90	0.85	4.8	4.8	5.0	4.9
500	G1	33.7	33.2	33.1	33.3	0.86	0.92	0.87	0.88	5.4	5.6	5.2	5.4
	G2	32.8	32.5	32.2	32.5	0.77	0.72	0.80	0.76	4.6	4.3	4.3	4.4
	G3	31.3	31.1	31.7	31.4	0.73	0.71	0.68	0.71	3.1	3.0	2.8	3.0
	S	30.2	29.9	29.4	29.8	0.55	0.65	0.49	0.56	3.5	3.2	3.3	3.3

**Table 2:** Reopening pressure measured and calculated by Equations (5) and (18) at various temperatures and lithologies.

T/°C	Type	*P*_r_/MPa Equation (5)				*P*_r_/MPa Equation (18)				*P*_r_/MPa (Measured)			
		No.1	No.1	No.1	Avg.	No.1	No.1	No.1	Avg.	No.1	No.1	No.1	Avg.
100	G1	30.7	30.4	30.6	30.6	28.8	28.9	29.5	29.1	27.8	28.3	28.7	28.3
	G2	30.6	30.4	29.9	30.3	28.4	28.0	28.0	28.1	27.4	27.1	27.8	27.4
	G3	29.7	30.4	29.5	29.9	26.9	28.0	26.6	27.2	26.7	27.1	26.5	26.8
	S	29.1	28.5	29.8	29.1	24.2	23.8	24.6	24.2	24.0	24.2	23.6	23.9
300	G1	29.6	30.1	30.2	30.0	25.7	26.9	26.4	26.3	25.2	26.1	25.3	25.5
	G2	29.8	29.9	29.3	29.7	25.8	26.3	25.1	25.7	25.1	25.6	24.9	25.2
	G3	29.5	29.9	29.5	29.6	24.9	25.4	25.4	25.2	24.4	24.5	25.0	24.6
	S	28.8	28.1	28.3	28.4	22.2	21.9	22.4	22.2	21.8	22.3	22.7	22.3
500	G1	28.3	27.6	27.9	27.9	22.1	21.9	21.8	21.9	22.4	22.9	22.5	22.6
	G2	28.2	28.2	27.9	28.1	21.4	21.1	21.3	21.3	21.6	21.2	21.9	21.6
	G3	28.2	28.1	28.9	28.4	21.2	20.9	21.5	21.2	21.3	21.1	20.8	21.1
	S	26.7	26.7	26.1	26.5	18.4	19.1	17.4	18.3	19.7	20.6	19.2	19.8

## Data Availability

The data used to support the findings of this study are included within the article.

## References

[1] Klee G., Bunger A., Meyer G., Rummel F., Shen B. (2011). In Situ Stresses in Borehole Blanche-1/South Australia Derived from Breakouts, Core Discing and Hydraulic Fracturing to 2 km Depth. Rock Mech. Rock Eng..

[2] Pruess K. (2006). Enhanced geothermal systems (EGS) using CO_2_ as working fluid—A novel approach for generating renewable energy with simultaneous sequestration of carbon. Geothermics.

[3] Garcia J., Hartline C., Walters M., Wright M., Rutqvist J., Dobson P.F., Jeanne P. (2016). The Northwest Geysers EGS Demonstration Project, California. Geothermics.

[4] Ito T., Evans K., Kawai K., Hayashi K. (1999). Hydraulic fracture reopening pressure and the estimation of maximum horizontal stress. Int. J. Rock Mech. Min. Sci..

[5] Rahman M.M., Rahman M.K., Rahman S.S. (2001). An integrated model for multiobjective design optimization of hydraulic fracturing. J. Pet. Sci. Eng..

[6] Aghighi M.A., Rahman S.S. (2010). Initiation of a secondary hydraulic fracture and its interaction with the primary fracture. Int. J. Rock Mech. Min. Sci..

[7] Rutqvist J., Tsang C., Stephansson O., Tsang C., Myer L. (2000). Uncertainty in the maximum principal stress estimated from hydraulic fracturing measurements due to the presence of the induced fracture. Int. J. Rock Mech. Min. Sci..

[8] Sugawara K., Obara Y. (1999). Draft ISRM suggested method for in situ stress measurement using the compact conical-ended borehole overcoring (CCBO) technique. Int. J. Rock Mech. Min. Sci..

[9] Wang D., Dahi Taleghani A., Yu B., Wang M., He C. (2022). Numerical simulation of fracture propagation during refracturing. Sustainability.

[10] Cornet F.H., Doan M.L., Fontbonne F. (2003). Electrical imaging and hydraulic testing for a complete stress determination. Int. J. Rock Mech. Min. Sci..

[11] Li Q., Ma D., Zhang Y.D., Liu Y., Ma Y.J. (2021). Insights into controlling factors of pore structure and hydraulic properties of broken rock mass in a geothermal reservoir. Lithosphere.

[12] Ito T., Igarashi A., Kato H., Ito H., Sano O. (2006). Crucial effect of system compliance on the maximum stress estimation in the hydrofracturing method: Theoretical considerations and field-test verification. Earth Planets Space.

[13] Mohammadnejad T., Andrade J.E. (2016). Numerical modeling of hydraulic fracture propagation, closure and reopening using XFEM with application to in-situ stress estimation. Int. J. Numer. Anal. Met..

[14] Lu C., Guo J., Liu L. (2016). A new calculation model for the stress field of hydraulic fracture propagation at the formation interface. Environ. Earth Sci..

[15] Leblond J. (1999). Crack paths in three-dimensional elastic solids. i: Two-term expansion of the stress intensity factors—Application to crack path stability in hydraulic fracturing. Int. J. Solids Struct..

[16] Zhang Z.B., Li X. (2016). The shear mechanisms of natural fractures during the hydraulic stimulation of shale gas reservoirs. Materials.

[17] Papanastasiou P. (1999). The effective fracture toughness in hydraulic fracturing. Int. J. Fracture.

[18] Petit J., Wibberley C.A.J., Ruiz G., Evans J.P., Treagus S.H. (1999). “Crack-seal”, slip; a new fault valve mechanism?. J. Struct. Geol..

[19] Shauer N., Duarte C.A. (2020). A generalized finite element method for three-dimensional hydraulic fracture propagation: Comparison with experiments. Eng. Fract. Mech..

[20] Dontsov E.V., Peirce A.P. (2015). An enhanced pseudo-3D model for hydraulic fracturing accounting for viscous height growth, non-local elasticity, and lateral toughness. Eng. Fract. Mech..

[21] Zhou Z., Jin Y., Zeng Y., Zhang X., Zhou J., Zhuang L., Xin S. (2020). Investigation on fracture creation in hot dry rock geothermal formations of China during hydraulic fracturing. Renew. Energ..

[22] Avanthi Isaka B.L., Ranjith P.G., Rathnaweera T.D. (2019). The use of super-critical carbon dioxide as the working fluid in enhanced geothermal systems (EGSs): A review study. Sustain. Energy Technol. Assess..

[23] Zhuang D., Yin T., Li Q., Wu Y., Tan X. (2022). Effect of injection flow rate on fracture toughness during hydraulic fracturing of hot dry rock (HDR). Eng. Fract. Mech..

[24] Li N., Zhang S., Wang H., Ma X., Zou Y., Zhou T. (2021). Effect of thermal shock on laboratory hydraulic fracturing in Laizhou granite: An experimental study. Eng. Fract. Mech..

[25] Zhang L., Yang L.C., Geng S.H., Ren S.R. (2021). Comparative experiment of wellbore self-circulation heat mining capacity with different heat-carrying fluids. Lithosphere.

[26] Kumari W.G.P., Ranjith P.G., Perera M.S.A., Li X., Li L.H., Chen B.K., Isaka B.L.A., De Silva V.R.S. (2018). Hydraulic fracturing under high temperature and pressure conditions with micro CT applications: Geothermal energy from hot dry rocks. Fuel.

[27] Feng F.P., Han X., Suo Y., Wang H.Y., Ye Q.Y., Duan Y.W. (2021). Study on synchronous propagation behavior of hydraulic fractures and cementing interfacial cracks during fracturing of shale horizontal wells. Lithosphere.

[28] Choi S., Jeon B., Lee S., Jeon S. (2019). Experimental study on hydromechanical behavior of an artificial rock joint with controlled roughness. Sustainability.

[29] Evans K.F., Cornet F.H., Hashida T., Hayashi K., Ito T., Matsuki K., Wallroth T. (1999). Stress and rock mechanics issues of relevance to HDR/HWR engineered geothermal systems: Review of developments during the past 15 years. Geothermics.

[30] Feng G., Liu C.B., Wang J.L., Tao Y., Duan Z.P., Xiang W.N. (2022). Experimental study on the fracture toughness of granite affected by coupled mechanical-thermo. Lithosphere.

[31] Feng Y., Chen X., Xu X.F. (2014). Current status and potentials of enhanced geothermal system in China: A review. Renew. Sust. Energ. Rev..

[32] Moska R., Labus K., Kasza P. (2021). Hydraulic Fracturing in Enhanced Geothermal Systems—Field, Tectonic and Rock Mechanics Conditions—A Review. Energies.

[33] Zhang Y., Luo J., Feng J.Y. (2020). Characteristics of geothermal reservoirs and utilization of geothermal resources in the southeastern coastal areas of China. J. Groundw. Sci. Eng..

[34] Haimson B.C., Cornet F.H. (2003). ISRM suggested methods for rock stress estimation—Part 3: Hydraulic fracturing (HF) and/or hydraulic testing of pre-existing fractures (HTPF). Int. J. Rock Mech. Min. Sci..

[35] Mao R., Feng Z., Liu Z., Zhao Y. (2017). Laboratory hydraulic fracturing test on large-scale pre-cracked granite specimens. J. Nat. Gas. Sci. Eng..

[36] Clifton R.J., Simonson E.R., Jones A.H., Green S.J. (1976). Determination of the Critical-Stress-Intensity Factor KIc From Internally Pressurized Thick-Walled Vessels. Exp. Mech..

[37] Bowie O.L., Freese C.E. (1972). Elastic analysis for a radial crack in a circular ring. Eng. Fract. Mech..

[38] Ghanbari A., Shams Rad S. (2015). Development of an empirical criterion for predicting the hydraulic fracturing in the core of earth dams. Acta Geotech..

[39] Abou-Sayed A.S., Brechtel C.E., Clifton R.J. (1978). In situ stress determination by hydrofracturing; a fracture mechanics approach. J. Geophys. Res..

[40] Desayi P., Veerappan M. (1972). A new indirect tension test for concrete and other brittle materials. Mater. Constr..

[41] Hubbert M.K., Willis D.G. (1957). Mechanics of hydraulic fracturing. Pet. Trans. AIME.

[42] England A.H., Green A.E. (1963). Some two-dimensional punch and crack problems in classical elasticity. Math. Proc. Camb. Philos. Soc..

[43] Tan P., Jin Y., Pang H. (2021). Hydraulic fracture vertical propagation behavior in transversely isotropic layered shale formation with transition zone using XFEM-based CZM method. Eng. Fract. Mech..

[44] Nordgren R.P. (1972). Propagation of a vertical hydraulic fracture. Soc. Pet. Eng. J..

[45] Yue K., Lee H.P., Olson J.E., Schultz R.A. (2020). Apparent fracture toughness for LEFM applications in hydraulic fracture modeling. Eng. Fract. Mech..

[46] Zhuang D., Yin T., Li Q., Wu Y., Chen Y., Yang Z. (2022). Fractal fracture toughness measurements of heat-treated granite using hydraulic fracturing under different injection flow rates. Theor. Appl. Fract. Mec..

[47] Dontsov E.V., Peirce A.P. (2016). Comparison of toughness propagation criteria for blade-like and pseudo-3D hydraulic fractures. Eng. Fract. Mech..

[48] Imad A., Wilsius J., Abdelaziz M.N., Mesmacque G. (2003). Experiments and numerical approaches to ductile tearing in an 2024-T351 aluminium alloy. Int. J. Mech. Sci..

[49] Cheng L., Luo Z., Yu Y., Zhao L., Zhou C. (2019). Study on the interaction mechanism between hydraulic fracture and natural karst cave with the extended finite element method. Eng. Fract. Mech..

